# Freeze-Dried Banana Slices Carrying Probiotic Bacteria

**DOI:** 10.3390/foods12122282

**Published:** 2023-06-06

**Authors:** Carolina M. Niro, Giovana M. N. Mendonça, Lucca R. Paulino, Viviane F. Soares, Henriette M. C. Azeredo

**Affiliations:** 1 Graduate Program in Biotechnology, Federal University of São Carlos (UFSCar), São Carlos 13565-905, Brazil; carolina.niro@estudante.ufscar.br; 2 Graduate Program in Food, Nutrition and Food Engineering, São Paulo State University (UNESP), Araraquara 14800-903, Brazil; giovana.mendonca@unesp.br; 3 São Carlos School of Engineering, University of São Paulo (USP), São Carlos 13566-590, Brazil; lucca.ribeiro@usp.br; 4 Embrapa Instrumentation, São Carlos 13560-970, Brazil; viviane.soares@embrapa.br

**Keywords:** polysaccharides, edible coatings, tropical fruits

## Abstract

Findings on diet–health relationships have induced many people to adopt healthier diets, including the substitution of energy-dense snacks with healthier items, e.g., those containing probiotic microorganisms. The aim of this research was to compare two methods to produce probiotic freeze-dried banana slices—one of them consisting of impregnating slices with a suspension of probiotic *Bacillus coagulans*, the other based on coating the slices with a starch dispersion containing the bacteria. Both processes resulted in viable cell counts above 7 log ufc.g^−1^, although the presence of the starch coating prevented a significant loss in viability during freeze-drying. The coated slices were less crispy than the impregnated ones, according to the shear force test results. However, the sensory panel (with more than 100 panelists) did not perceive significant texture differences. Both methods presented good results in terms of probiotic cell viability and sensory acceptability (the coated slices being significantly more accepted than the non-probiotic control slices).

## 1. Introduction

Banana is one of the most produced fruits in the world. In 2021, about 125 million tons were produced [1]. Bananas are mostly consumed as fresh fruits, but they are also processed into flours, purees, jams, sauces, and snacks (mainly dehydrated bananas, such as banana figs and freeze-dried banana slices). People usually associate snacks with energy-dense food products high in sodium, sugar and/or fat; however, the consumption of healthier snacks has become a trend, including those based on fruits and vegetables and probiotic-enriched snacks [2]. Some studies have described the development of probiotic snacks based on fruits, including apples [3,4], strawberries [5], papayas [6], and bananas [7]. This is part of the effort to produce non-dairy probiotic food products, since most probiotic foods in the market are dairy, and thus not suitable for consumers with dietary restrictions to milk and derivatives [8].

However, stress factors during food processing and storage (including thermal, osmotic and oxidative stresses, dehydration, and shear forces) may impair the viability of probiotic microorganisms [9]. That is the main reason why spore-forming probiotic bacteria, due to their high resistance to heat, low pH and other environmental stresses, have been the microorganisms of choice in some studies [5,10,11]. The probiotic properties of *Bacillus coagulans* have been summarized elsewhere [12].

Dehydrated fruit slices may be incorporated with probiotics either by direct impregnation with a probiotic suspension [5,13,14], or by having an edible polysaccharide-based coating containing probiotics applied to them [4,5,6], which might help protect the probiotic, as well as potentially helping adhesion of the probiotics to the epithelial cells of the intestine by hydrogen bonding [15]. 

This study was carried out to obtain probiotic freeze-dried banana slices either by impregnating banana slices with a probiotic suspension or by coating them with a starch-based dispersion containing probiotics prior to freeze drying, comparing the products of the two processes.

## 2. Materials and Methods

### 2.1. Processing of Probiotic Banana Slices

Two probiotic formulations were prepared, namely a probiotic impregnating suspension and a probiotic coating. The impregnation suspension was prepared by adding 2.5 g of freeze-dried *B. coagulans* BC4 50 MLD spores (from lot C235515A) standardized with maltodextrin, approximately 10^11^ cfu g^−1^, as provided by Sacco (Cadorago, Italy), into 500 mL of distilled water (previously autoclaved at 121 °C for 15 min, then cooled back to 25 °C). The probiotic coating was prepared by suspending 10 g of corn starch in 500 mL of distilled water with 3 g of glycerol. The dispersion was kept at 85 °C for 45 min under stirring (150 rpm) for starch gelatinization, then autoclaved at 121 °C for 15 min, cooled back to 25 °C, and supplemented with 2.5 g of *B. coagulans* spores.

Overripe ‘Prata’ bananas (*Musa sapientum*) were purchased from a single supplier in São Carlos, SP, Brazil, washed with neutral detergent, rinsed, disinfected via 5 min immersion in chlorinated water (100 mg L^−1^), rinsed, peeled, cut into 5 mm-thick slices, and then blanched in a boiling citric acid 1 wt% solution for 1 min (in order to inactivate enzymes, including polyphenol oxidase, and thus avoid enzymatic browning). The slices were separated into four groups, each one containing 60 slices. For each group, the slices received one of four treatments, namely: COAT-Pro (immersion for 1 min in the starch-based probiotic coating), IMP-Pro (impregnation for 1 min with the probiotic impregnating suspension), COAT (immersion for 1 min in a starch-based coating without probiotics) and C (control—immersion for 1 min in 500 mL of previously autoclaved distilled water). The slices were then pre-frozen in an ultra-freezer at −25 °C for 24 h, then freeze-dried in a Liotop L101 freeze-dryer (Liotop, São Carlos, SP, Brazil) for 6 days. 

All equipment and glassware used in the experiment were previously either aseptized with ethanol 70 vol% or autoclaved (121 °C, 15 min) to avoid contamination.

### 2.2. Characterization of Probiotic Banana Slices

#### 2.2.1. Viable Cell Counts

From each treatment, three 1-g samples were taken before and after freeze-drying for viable cell counting. The samples were homogenized with 9 mL of a previously sterilized saline solution (0.85 wt% NaCl), and then subjected to serial dilutions (up to 10^−5^), plated (in triplicate) on tryptone glucose yeast extract (TGY) agar by the drop plate method, and incubated at 37 °C for 48 h. The remaining slices were pre-frozen in an ultra-freezer at −25 °C for 24 h, then freeze-dried in a Liotop L101 freeze-dryer (Liotop, São Carlos, SP, Brazil) for 6 days. 

#### 2.2.2. Shear Force

The shear force was measured in a texture analyzer (Stable Micro Systems Ltd., Godalming, UK) using a Knife Edge blade and slotted base (HDP/BS) at 2 mm s^−1^, to determine the force required to cross-cut the slices, simulating the action of incisor teeth on a first bite (Paula & Conti-Silva, 2014). The test was performed with ten replicates (each replicate being a banana slice). The results are expressed as the peak force in Newtons (N).

#### 2.2.3. Surface Color

The surface color was determined using a Chroma Meter CR 410 (Konica Minolta, Ramsey, NJ, USA), with 24 replicates (measured at two points of each face of 6 slices). After measuring the L*, a*, and b* values, chromaticity (C*) and Hue angle (*h°*) were determined using the following equations:

(1) $$C^{*}=\sqrt{a^{*}{}^{2}+b^{*}{}^{2}}\;$$

(2) $$h^{{}^{\circ}}=tan^{-1}(\frac{b^{*}}{a^{*}})$$

#### 2.2.4. Scanning Electron Microscopy (SEM)

Sections (10 mm^2^, 1-mm thick) were dissected from the banana slice surfaces for SEM. The specimens were fixed to aluminum stubs using conductive carbon tape and sputter-coated with a 10 nm-thick gold layer using an ACE600 Sputter Coater (Leica Microsystems, Wetzlar, Germany). Cross-sections were obtained by immersing slices into liquid N_2_ and fracturing them. The samples were fixed to aluminum stubs (with the fractured surface facing upward) using conductive carbon tape and sputter-coated with a 10 nm-thick gold layer. Images were taken with a JSM 6510 (Jeol, Tokyo, Japan) microscope at 5 kV, with 1000× magnification. 

#### 2.2.5. Sensory Evaluation

A sensory evaluation was conducted in individual booths with white light, with 107 panelists aged between 18 and 60+ years. The panelists received two-slice samples of each of three treatments (namely, COAT-Pro, IMP-Pro, and C) in randomized order, each sample being codified with randomized 3-digit numbers. The reason why only three (instead of four) treatments were included in the sensory test was that the probiotics themselves were assumed not to change the sensory properties, whereas the method of incorporating them was. Therefore, COAT-Pro and IMP-Pro were analyzed, and the control was added as well for comparison. The panelists were required to fill out an online form containing two questions for each sample. The first question was on overall acceptance, in which they were asked to respond how much they liked each sample, on a 9-point structured hedonic scale (from 1—“extremely disliked” to 9—“extremely liked”). The second question was about the crispiness of the sample, on a 5-point structured ideal scale ranging from −2 (“much less crispy than ideal”) to +2 (“much crispier than ideal”), 0 representing the ideal crispiness. The averages of both the acceptance test and the crispiness test were compared using Tukey tests. The study was approved by the Research Ethics Committee of UFSCar (Federal University of São Carlos, CAAE n. 27815920.7.0000.5504).

## 3. Results and Discussion

**Figure 1:**
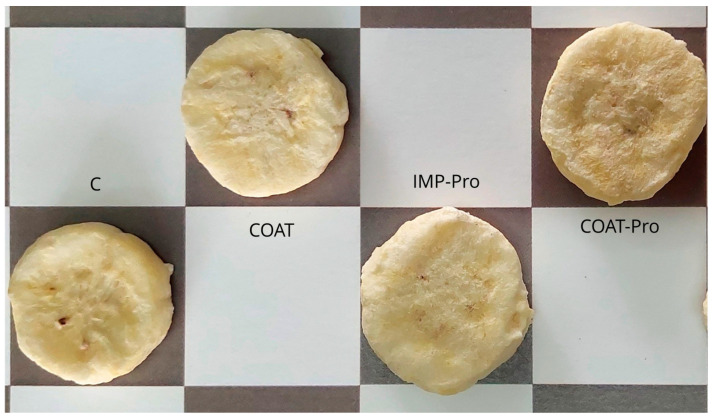
Photograph of banana slices from different treatments. C: control (no probiotics or coatings). COAT: slice with a starch–based coating without probiotics. IMP-Pro: slice impregnated with probiotics. COAT-Pro: slice with a starch–based coating containing probiotics.

**Figure 2:**
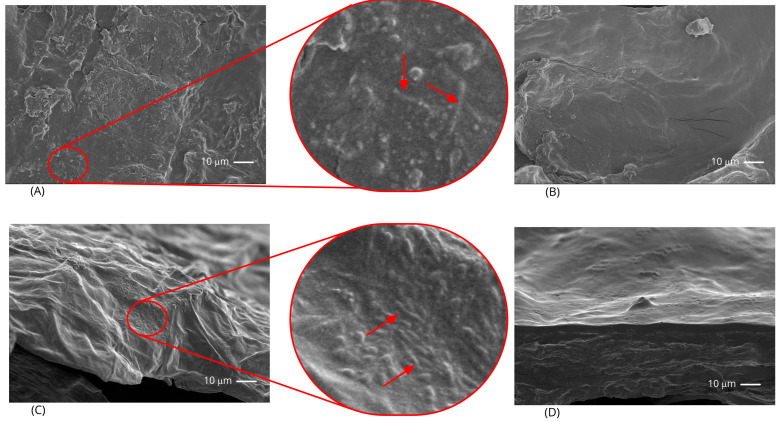
SEM micrographs of probiotic banana slices. (**A**,**C**) slices impregnated with probiotics (treatment IMP-Pro), surface and cross-section, respectively. The arrows indicate probable bacterial structures. (**B**,**D**) slices with starch-based coatings containing probiotics (treatment COAT-Pro), surface and cross-section, respectively. All micrographs were taken at 5 kV with a magnification of 1000× *g*.

The freeze-dried banana slices presented a light color, very similar to that of fresh slices (Figure 1), as expected from previously blanched freeze-dried slices. They were also very similar to each other, irrespective of treatment. SEM micrographs of the probiotic-containing slices (Figure 2) showed irregular rough structures (mainly on the surface of impregnated slices). In addition, the impregnated slices revealed the presence of bacteria on their surfaces, while the coated slices did not, probably because the bacteria were embedded into the starch matrix (since they were added into the coating-forming dispersion). 

Viable cell counts of the slices before and after freeze drying were compared. Those of the slices impregnated with probiotics were reduced after freeze drying, while those of the starch–probiotic-coated slices were not significantly reduced (Table 1), indicating that the starch coating provided some protection to the bacteria, corroborating studies showing protective effects of polysaccharides on probiotic microorganisms [16,17].

Table 2 presents the values of shear force and color (chromaticity and Hue angle) for banana slices from different treatments. There were no significant differences within treatments regarding the color attributes, i.e., neither the presence of coatings nor that of probiotics changed the instrumental color, corroborating the appearance similarities shown in Figure 1. The shear force of the coated slices was significantly higher than that of the uncoated ones, indicating that the presence of a coating increased the force required to fracture the slices; that is to say, coated slices became less crispy. On the other hand, the sensory panel did not perceive significant differences in crispiness within treatments (Table 3), indicating that the presence of the starch coating did not impair the sensory crispiness. Additionally, although the COAT-Pro and IMP-Pro treatments did not receive significantly different acceptance rates, the COAT-Pro samples were more accepted than the control, which was unexpected. While the samples were exposed for the panelists to evaluate them, the banana slices probably absorbed moisture from the surrounding environment, but the starch coating may have reduced such moisture absorption, causing the coated banana slices to be perceived as crispier than the uncoated ones, since the coating acts as a barrier to control the absorption of water vapor [18]. Similarly, freeze-dried banana slices pretreated with a quince seed mucilage coating were more accepted than control uncoated ones [19]. 

## 4. Conclusions

Probiotic banana slices were successfully obtained either by impregnating them with a *Bacillus coagulans* suspension or by coating them with a starch dispersion containing the bacteria in advance of freeze-drying. The presence of the starch coating prevented a significant loss in cell viability upon freeze-drying, but both processes resulted in viable cell counts above 7 log ufc.g^−1^. The instrumental texture (shear force) test indicated that the coated slices were less crispy than the impregnated ones but, according to the sensory test, the panelists did not perceive differences in texture (in terms of ideal crispiness) between impregnated and coated slices. The coated slices were as well accepted as the impregnated ones, but more accepted than the non-probiotic control slices. Both methods (impregnation and coating) are simple and present satisfactory results, in terms of both probiotic viability and sensory acceptability.

## Figures and Tables

**Table 1:** Viable probiotic cell counts (on a dry basis) in banana slices from different treatments, before and after freeze drying.

Treatment	Viable Cell Counts (log cfu g^−1^)	
	Before Freeze Drying	After Freeze Drying
C	n.d.	n.d.
COAT	n.d.	n.d.
IMP-Pro	7.92 ± 0.04 (*)	7.44 ± 0.14 (*)
COAT-Pro	7.95 ± 0.25	7.75 ± 0.17

C: control (banana slices not containing probiotics or coatings). COAT: banana slices with starch-based coatings without probiotics. IMP-Pro: banana slices impregnated with probiotics. COAT-Pro: banana slices with starch-based coatings containing probiotics. n.d.: non-detectable. Values in the same row followed by asterisks were significantly different (*t*-test, *p* < 0.05).

**Table 2:** Shear force and color attributes of freeze-dried banana slices from different treatments.

Treatment	Chromaticity	Hue (°)	Shear Force (N)
C	22.04 ± 4.26	−86.78 ± 1.08	73.89 ± 10.12 (b)
COAT	19.91 ± 1.76	−85.98 ± 1.17	113.60 ± 28.10 (a)
IMP-Pro	20.35 ± 1.64	−86.28 ± 0.92	71.28 ± 16.18 (b)
COAT-Pro	22.15 ± 2.37	−86.18 ± 0.68	115.12 ± 13.77 (a)

C: control (banana slices not containing probiotics or coatings). COAT: banana slices with starch-based coatings without probiotics. IMP-Pro: banana slices impregnated with probiotics. COAT-Pro: banana slices with starch-based coatings containing probiotics. Values in the same column followed by at least one common letter (or not followed by letters) were not significantly different (Tukey, *p* > 0.05).

**Table 3:** Results of the sensory evaluation.

Treatment	Overall Acceptance	Crispiness
C	6.19 ± 1.74 (b)	−0.53 ± 0.96
IMP-Pro	6.58 ± 1.73 (ab)	−0.44 ± 0.72
COAT-Pro	6.88 ± 1.72 (a)	−0.28 ± 0.78

Overall acceptance: values expressed on a 9-point structured hedonic scale (from 1—“extremely disliked” to 9—“extremely liked”). Crispiness: values expressed on a 5-point structured ideal scale (from −2—“much less crispy than ideal” to +2—“much crispier than ideal”), 0 representing the ideal crispiness. C: control (banana slices not containing probiotics or coatings). IMP-Pro: banana slices impregnated with probiotics. COAT-Pro: banana slices with starch-based coatings containing probiotics. Values in the same column followed by at least one common letter (or not followed by letters) were not significantly different (Tukey, *p* > 0.05).

## Data Availability

The data presented in this study are available on request from the corresponding author. The data are not publicly available since they contain information that could compromise research participant privacy.
